# Syk/JNK/AP-1 Signaling Pathway Mediates Interleukin-6-Promoted Cell Migration in Oral Squamous Cell Carcinoma

**DOI:** 10.3390/ijms15010545

**Published:** 2014-01-06

**Authors:** Jing-Yuan Chuang, Yuan-Li Huang, Wei-Lin Yen, I-Ping Chiang, Ming-Hsui Tsai, Chih-Hsin Tang

**Affiliations:** 1 Department of Medical Laboratory Science and Biotechnology, China Medical University, Taichung 402, Taiwan; E-Mails: alise9042@yahoo.com.tw (J.-Y.C.); wielin@pchome.com.tw (W.-L.Y.); 2 Department of Biotechnology, College of Health Science, Asia University, Taichung 402, Taiwan; E-Mail: yuanli@asia.edu.tw; 3 Department of Pathology, China Medical University Hospital, Taichung 402, Taiwan; E-Mail: d6868@mail.cmuh.org.tw; 4 Department of Otolaryngology, China Medical University Hospital, Taichung 402, Taiwan; E-Mail: minghsui@mail.cmuh.org.tw; 5 Graduate Institute of Basic Medical Science, China Medical University, Taichung 402, Taiwan; 6 Department of Pharmacology, School of Medicine, China Medical University, Taichung 402, Taiwan

**Keywords:** Interleukin-6 (IL-6), oral squamous cell carcinoma (OSCC), Intercellular adhesion molecule-1 (ICAM-1), Syk, c-Jun *N*-terminal kinase (JNK)

## Abstract

Oral squamous cell carcinoma (OSCC) typically migrates and metastasizes. Interleukin-6 (IL-6) is a multifunctional cytokine associated with disease status and cancer outcomes. The effect of IL-6 on human OSCC cells, however, is unknown. Here, we showed that IL-6 increased cell migration and Intercellular adhesion molecule-1 (ICAM-1) expression in OSCC cells. Pretreatment of OSCC cells with IL-6R monoclonal antibody (mAb) significantly abolished IL-6-induced cell migration and ICAM-1 expression. By contrast, IL-6-mediated cell motility and ICAM-1 upregulation were attenuated by the Syk and c-Jun *N*-terminal kinase (JNK) inhibitors. Stimulation of OSCC cells with IL-6 promoted Syk and JNK phosphorylation. Furthermore, IL-6 enhanced AP-1 activity, and the IL-6R mAb, Syk inhibitor, or JNK inhibitor all reduced IL-6-mediated c-Jun phosphorylation, c-Jun binding to the ICAM-1 promoter, and c-Jun translocation into the nucleus. Our results indicate that IL-6 enhances the migration of OSCC cells by increasing ICAM-1 expression through the IL-6R receptor and the Syk, JNK, and AP-1 signal transduction pathways.

## 1. Introduction

Oral squamous cell carcinoma (OSCC), the most common head and neck cancer, represents 1%–2% of all human malignancies and is characterized by poor prognosis and a low survival rate. OSCC has been reported to migrate into the maxillary and mandibular bones [1,2] and has a potent capacity to invade locally and metastasize distantly [3,4]. Hence, a decrease in its ability to invade and metastasize is a goal in the development of effective adjuvant therapy.

Interleukin (IL)-6, originally identified as a T cell-derived cytokine that induces the final maturation of B cells into antibody-producing cells [5], exhibits multiple biological activities that differ widely among various types of tissues and cells. IL-6 has been reported to enhance or inhibit the proliferation of carcinoma cells [6–9]. Furthermore, a variety of malignant tumors, including OSCC and adenocarcinomas were shown to contain or synthesize IL-6, and autocrine growth stimulation has been suggested as a possible mechanism for the action of IL-6 [10–12]. Recent studies have documented that IL-6 enhances cell migration in human chondrosarcoma and osteosarcoma [13,14].

Invasion and metastasis of tumors are critical steps in determining the aggressive phenotype of human cancers. Mortality in cancer patients principally results from the metastatic spread of cancer cells to distant organs [15,16]. To facilitate cell motility, invading cells must alter their cell-cell adhesion properties, rearrange the extracellular matrix environment, suppress anoikis, and reorganize their cytoskeletons [17]. Several cell adhesion molecules have been reported to be involved in tumor progression and metastasis such as integrin, cadherin, and immunoglobulin superfamilies [16,18]. Intercellular adhesion molecule-1 (ICAM-1, also called CD54), a member of the immunoglobulin supergene family, is an inducible surface glycoprotein that mediates adhesion-dependent cell-to-cell interactions [19,20]. ICAM-1 has been reported to mediate the migration of leukocytes from the capillary bed into the tissue [21]. On the other hand, ICAM-1 also promotes the movement of cells through the ECM [21]. A recent study indicated that ICAM-1 plays an important role during lung cancer invasion [22]. Pretreatment with ICAM-1 antibody or transfection with antisense ICAM-1 has been reported to reduce the migration of breast cancer cells [23]. Furthermore, ICAM-1 upregulation has been reported to play an important role during OSCC metastasis [24–26]. Therefore, ICAM-1 might play a critical role in tumorigenesis, and its disruption may prevent metastasis.

Previous studies confirmed that IL-6 is important in the metastasis of human cancer cells [14,27]. ICAM-1 is a potent metastatic factor that mediates cancer migration and metastasis. Although a role for IL-6 in metastasis has been implicated in some cancer cells, the signaling pathway for IL-6 in cell motility and ICAM-1 expression in human OSCC has not been extensively studied. In this study, we examined the intracellular signaling pathway involved in IL-6-induced ICAM-1 expression and tumor migration in human OSCC. Here, we reported that the interaction between IL-6 and IL-6R activates the Syk, c-Jun N-terminal kinase (JNK), and activator protein (AP)-1 pathways, leading to upregulation of ICAM-1 expression and cell migration. Our results indicate that IL-6 is a crucial factor during the metastasis of OSCC cells.

## 2. Results

### 2.1. IL-6 Increases Migration in OSCC (Oral Squamous Cell Carcinoma) Cells

**Figure 1.**
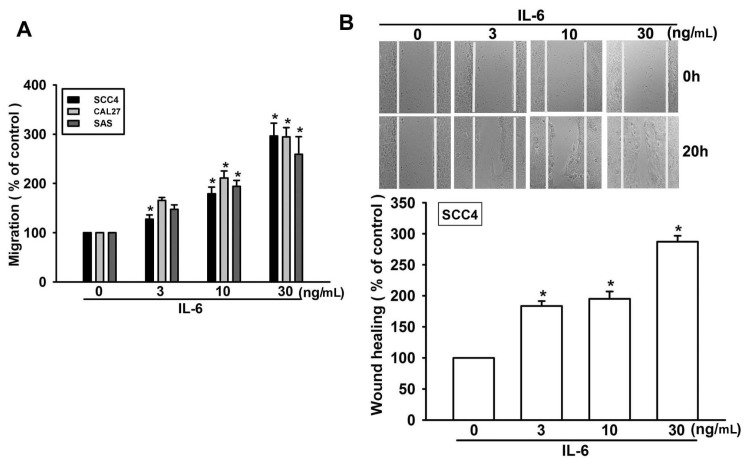
Interleukin (IL)-6 induces migration activity of human oral squamous cell carcinoma (OSCC) cells. (**A**) Cells were incubated with various concentrations of IL-6, and *in vitro* migration activity was measured using the Transwell assay after 24 h (*n* = 4); and (**B**) SCC4 cells were incubated with IL-6 for 24 h, and a wound-scratching assay was performed (*n* = 5). Results are expressed as the mean ± standard error of the mean (SEM); *, *p* < 0.05 compared with the control.

It has been reported that IL-6 stimulates the directional migration and invasion of human cancer cells [14,27]. However, the effect of IL-6 on the migration of OSCC cells is mostly unknown. To identify a link between IL-6 expression and OSCC migration, we first examined the migratory activity of human OSCC cells using the Transwell assay. Stimulation of OSCC cells (SCC4, SAS, and CAL27 cells) with IL-6 promoted cell migration in a dose-dependent manner (Figure 1A). In addition, IL-6 also increased wound healing migration activity in SCC4 cells (Figure 1B). Hence, IL-6 promotes cell migration in human OSCC cells.

### 2.2. Involvement of ICAM-1 in IL-6-Directed Cell Migration of OSCC Cells

**Figure 2.**
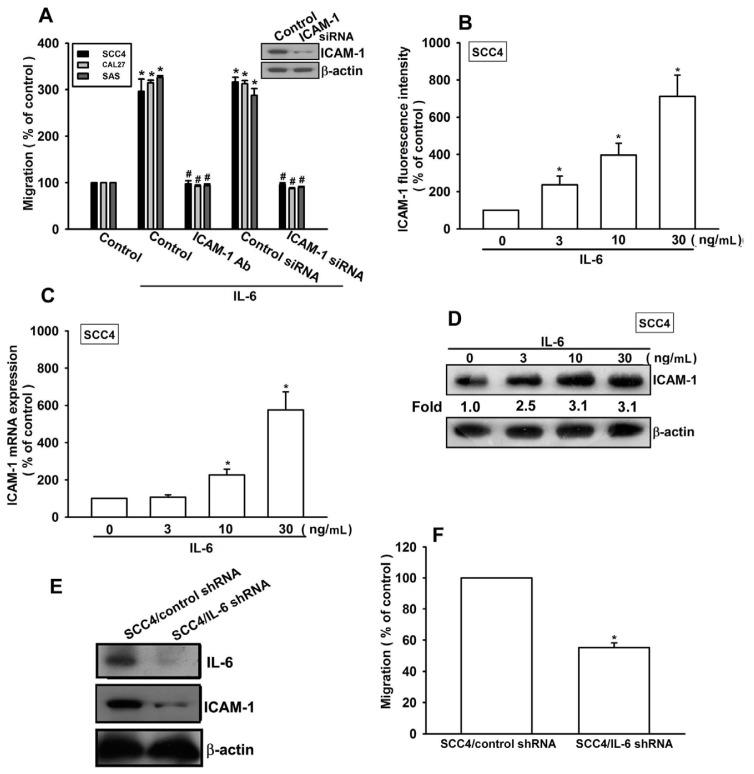
IL-6 increases cell migration by upregulating intercellular adhesion molecule-1 (ICAM-1). (**A**) Cells were pretreated for 30 min with ICAM-1 mAb (10 μg/mL) or transfected with ICAM-1 small interfering RNA (siRNA) for 24 h, followed by stimulation with IL-6 (30 ng/mL). *In vitro* migration activity was measured with the Transwell assay (*n* = 5); (**B**–**D**) SCC4 cells were incubated with IL-6 for 24 h, and ICAM-1 expression was examined by flow cytometry, quantitative real-time polymerase chain reaction (qPCR), and western blotting (*n* = 6); and (**E**,**F**) Protein levels and migratory activity of IL-6 and ICAM-1 in SCC4/control short hairpin RNA (shRNA) and SCC4/IL-6 shRNA cells were examined by western blotting and the Transwell assay (*n* = 5). Results are expressed as the mean ± SEM; *, *p* < 0.05 compared with the control; #, *p* < 0.05 compared with the IL-6-treated group.

A previous report indicated that ICAM-1 mediates the migration and metastasis of OSCC cells [24]. We therefore examined whether ICAM-1 is involved in IL-6-induced migration of OSCC cells. Pretreatment of cells with ICAM-1 monoclonal antibody (mAb) or transfection of cells with ICAM-1 siRNA abolished IL-6-induced cell migration (Figure 2A). By contrast, incubation of cells with IL-6 increased the cell surface, mRNA, and protein expression of ICAM-1 (Figure 2B–D). To confirm that IL-6 mediates cell migration and ICAM-1 expression in human OSCC cells, SCC4 cells expressing IL-6 shRNA were established. IL-6 expression in stable transfectants was compared by western blotting. Expression of IL-6 was dramatically inhibited in SCC4/IL-6 shRNA cells (Figure 2E). However, knockdown of IL-6 did not affect SCC4 cell growth (data not shown). The migratory ability of these transfectants was then analyzed using a Transwell migration assay. Knockdown of IL-6 expression inhibited the migratory ability of SCC4 cells (Figure 2F). In addition, IL-6 knockdown also reduced ICAM-1 expression in SCC4 cells (Figure 2E). These results indicate that IL-6 increases cell migration by upregulating ICAM-1 in human OSCC cells.

**Figure 3.**
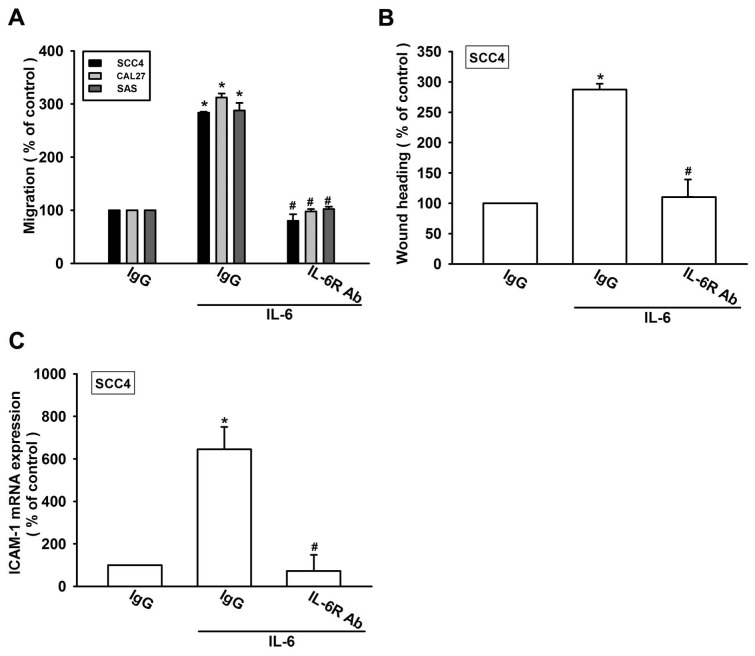
IL-6 and IL-6R interaction promotes cell migration and ICAM-1 expression. (**A**–**C**) Cells were pretreated with IL-6R monoclonal antibody (mAb) (10 μg/mL) for 30 min followed by stimulation with IL-6 (30 ng/mL) for 24 h. The *in vitro* migration activity and ICAM-1 expression were measured with the Transwell, wound healing, and qPCR assays (*n* = 5); *, *p* < 0.05 compared with the control; #, *p* < 0.05 compared with the IL-6-treated group.

IL-6 is known to affect tumor migration by binding to cell-surface IL-6R molecules [13,14]. Pretreating cells for 30 min with anti-IL-6R mAb significantly reduced IL-6-increased cell migration and ICAM-1 expression (Figure 3A–C). Thus, IL-6 increased cell migration and ICAM-1 expression in human OSCC cells via the IL-6R receptor.

### 2.3. Syk and c-Jun *N*-Terminal Kinase (JNK) Signaling Pathways are Involved in IL-6-Mediated ICAM-1 Upregulation and Cell Motility of OSCC Cells

**Figure 4.**
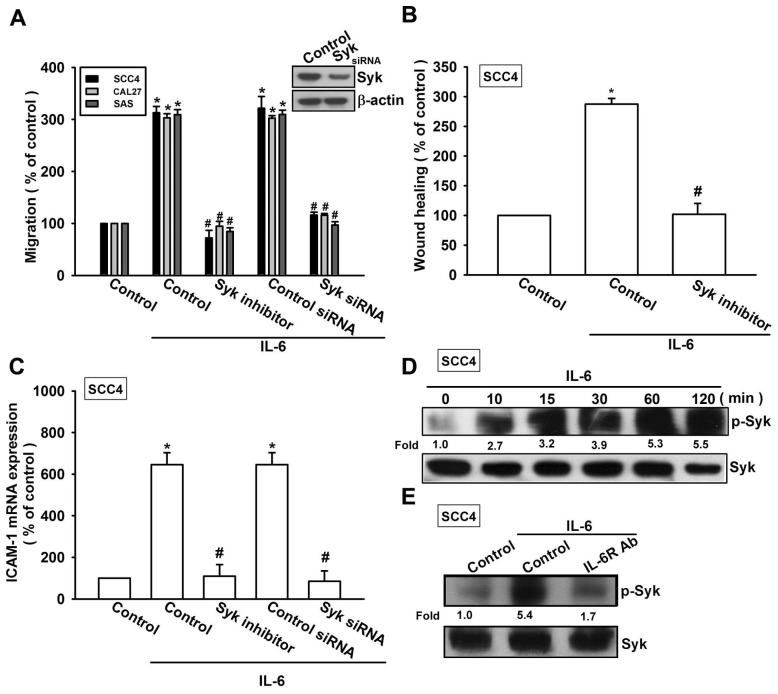
Syk is involved in IL-6-induced migration and ICAM-1 expression. (**A**–**C**) Cells were pretreated for 30 min with Syk inhibitor (10 μM) or transfected with Syk siRNA for 24 h and stimulated with IL-6 (30 ng/mL) for 24 h. *In vitro* migration and ICAM-1 expression were measured using the Transwell, wound healing, and qPCR assays (*n* = 5); (**D**) SCC4 cells were incubated with IL-6 (30 ng/mL) for the indicated time intervals, and Syk phosphorylation was examined by western blotting (*n* = 5); and (**E**) SCC4 cells were pretreated for 30 min with IL-6R mAb and stimulated with IL-6 (30 ng/mL) for 15 min; Syk phosphorylation was determined by western blotting (*n* = 4). Results are expressed as the mean ± SEM; *, *p* < 0.05 compared with the control; #, *p* < 0.05 compared with the IL-6-treated group.

**Figure 5.**
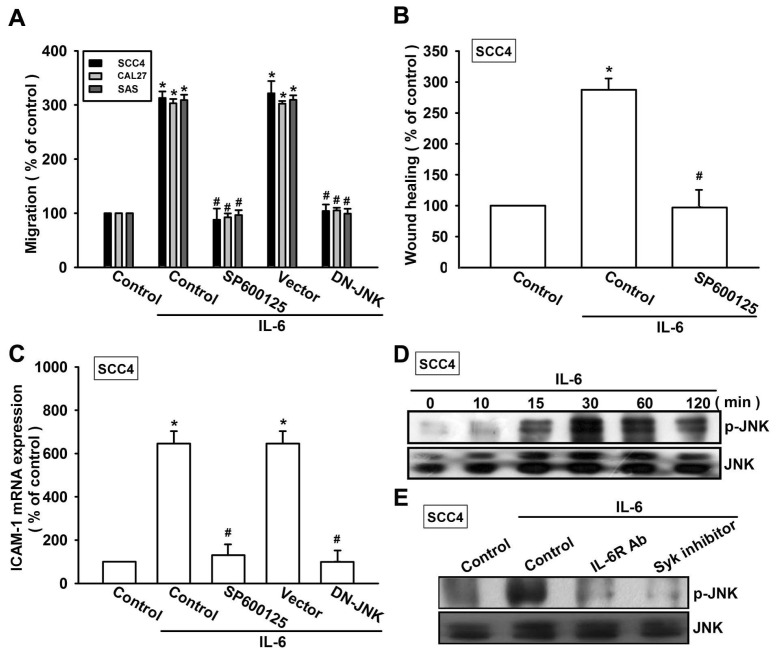
JNK is involved in IL-6-induced migration and ICAM-1 expression. (**A**–**C**) Cells were pretreated for 30 min with SP600125 (3 μM) or transfected with the JNK mutant for 24 h and stimulated with IL-6 (30 ng/mL) for 24 h. *In vitro* migration and ICAM-1 expression were measured using the Transwell, wound healing, and qPCR assays (*n* = 5); (**D**) SCC4 cells were incubated with IL-6 (30 ng/mL) for the indicated time intervals, and JNK phosphorylation was examined by western blotting (*n* = 5); and (**E**) SCC4 cells were pretreated for 30 min with IL-6R mAb or the Syk inhibitor and stimulated with IL-6 (30 ng/mL) for 30 min; Syk phosphorylation was determined by western blotting (*n* = 4). Results are expressed as the mean ± SEM; *, *p* < 0.05 compared with the control; #, *p* < 0.05 compared with the IL-6-treated group.

Syk-dependent JNK activation has been reported to mediate VCAM-1 expression [28]. We therefore hypothesized that the Syk and JNK signaling pathways are involved in IL-6-directed cell migration activity in OSCC cells. Our results showed that the IL-6-induced migration ability and ICAM-1 upregulation of OSCC cells were significantly reduced by pretreating the cells with Syk inhibitor (Figure 4A–C). In addition, transfection of cells with Syk siRNA also inhibited IL-6-induced motility and ICAM-1 expression (Figure 4A–C). We next directly measured the phosphorylation of Syk in response to IL-6. Stimulation of SCC4 cells with IL-6 increased Syk phosphorylation (Figure 4D). In addition, pretreating the cells with IL-6R mAb abolished IL-6-enhanced Syk phosphorylation (Figure 4E). Thus, IL-6 appears to act through IL-6R and the Syk-dependent signaling pathway to enhance cell migration and ICAM-1 expression in human OSCC cells. Next, we investigated the role of JNK in mediating IL-6-induced cell migration and ICAM-1 expression using the specific JNK inhibitor SP600125. Pretreatment of cells for 30 min with SP600125 or transfection of cells for 24 h with the JNK mutant reduced IL-6-induced increases in cell motility and ICAM-1 expression (Figure 5A–C). To directly confirm the critical role of JNK in IL-6-mediated cell migration, we measured the level of JNK phosphorylation in response to IL-6. Stimulation of SCC4 cells with IL-6 resulted in time-dependent phosphorylation of JNK (Figure 5D). We next evaluated the relationship between IL-6R, Syk, and JNK in the IL-6-mediated signaling pathway. Incubating the cells with IL-6R mAb or Syk inhibitor diminished IL-6-increased JNK phosphorylation (Figure 5E). Based on these results, IL-6 appears to act via the IL-6R receptor and the Syk and JNK-dependent signaling pathways to enhance cell migration and ICAM-1 expression in human OSCC cells.

### 2.4. Involvement of AP-1 in IL-6-Induced Cell Migration and ICAM-1 Expression

**Figure 6.**
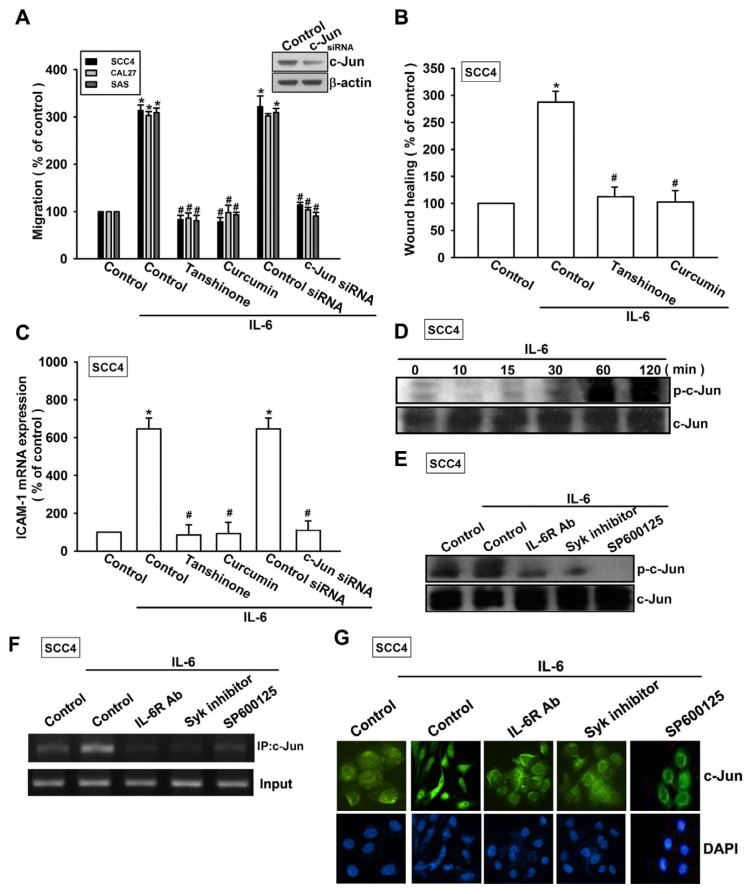
Activator protein 1 (AP-1) is involved in IL-6-mediated migration in human OSCC cells. (**A**–**C**) Cells were pretreated for 30 min with curcumin (10 μM) and tanshinone (10 μM) or transfected for 24 h with c-Jun siRNA followed by stimulation with IL-6 for 24 h. *In vitro* migration and ICAM-1 expression were measured using the Transwell, wound healing, and qPCR assays (*n* = 5); (**D**) SCC4 cells were incubated with IL-6 (30 ng/mL) for the indicated time intervals, and c-Jun phosphorylation was examined by western blotting (*n* = 4); and (**E**–**G**) SCC4 cells were pretreated for 30 min with IL-6R mAb, the Syk inhibitor, or SP600125 for 30 min followed by stimulation with IL-6 for 60 min. c-Jun phosphorylation, c-Jun binding to the AP-1 element, and c-Jun translocation into the nucleus were determined by western blotting, ChIP, and immunofluorocytochemistry (*n* = 5). Results are expressed as the mean ± SEM; *, *p* < 0.05 compared with the control; #, *p* < 0.05 compared with the IL-6-treated group.

As previously described, AP-1 transactivation is involved in cell migration and ICAM-1 expression in human OSCC cells [29]. To examine the role of the AP-1-binding site in IL-6-mediated cell motility and ICAM-1 expression, we used AP-1 inhibitors (curcumin and tanshinone). Pretreating cells with curcumin and tanshinone reduced IL-6-induced cell migration and ICAM-1 expression (Figure 6A–C). AP-1 activation was further evaluated by analyzing c-Jun phosphorylation and c-Jun translocation into the nucleus, and by conducting a chromatin immunoprecipitation (ChIP) assay. Transfection of cells with c-Jun siRNA suppressed IL-6-induced cell migration and ICAM-1 expression (Figure 6A,C). Incubating the cells with IL-6 promoted time-dependent phosphorylation of c-Jun (Figure 6D). In contrast, pretreating the cells with IL-6R, Syk inhibitor, or SP600125 abolished IL-6-mediated c-Jun phosphorylation (Figure 6E).

The AP-1 binding site between −284 and −279 is important for activation of the ICAM-1 gene [30]. We next investigated whether c-Jun binds to the AP-1 element in the ICAM-1 promoter after IL-6 stimulation. *In vivo* recruitment of c-Jun to the ICAM-1 promoter (−346 to −24) was assessed by ChIP. *In vivo* binding of c-Jun to the AP-1 element of the ICAM-1 promoter occurred after IL-6 stimulation (Figure 6F). Binding of c-Jun to the AP-1 element by IL-6 was attenuated by IL-6R, the Syk inhibitor, or SP600125 (Figure 6F). In addition, IL-6R, the Syk inhibitor, or SP600125 also reduced IL-6-increased c-Jun accumulation in the nucleus (Figure 6G). Taken together, these data suggest that activation of the IL-6R, Syk, and JNK pathways are required for IL-6-induced AP-1 activation and tumor metastasis in human OSCC cells.

## 3. Discussion

Efforts to understand the molecular biology of cancer cells in recent years have identified various molecular pathways that are altered in different cancers. This information is currently being exploited to develop potential therapies that target the molecules involved in these pathways. To achieve metastasis, cancer cells must evade multiple barriers and overcome specific cellular rules. Several discrete steps are discernible in the biological cascade leading to metastasis, including loss of cellular adhesion, increased motility and invasiveness, entry and survival into circulation, entrance into new tissue, and eventual colonization of a distant site [15]. The mechanism of metastasis is a complicated and multistage process; however, our study showed that IL-6 induces cell migration and expression of ICAM-1 in human OSCC cells. In addition, we found that ICAM-1 acts as a crucial transducer of cell signaling that regulates cell migration, whereas IL-6 acts as a critical mediator of metastasis activity of OSCC cells in the tumor microenvironment.

ICAM-1 is upregulated in response to a variety of cytokines and is associated with inflammatory and immune responses [21]. Several lines of evidence in this study showed that, in addition to its role in leukocyte adhesion and cancer cell invasion [31,32], ICAM-1 plays an important role in IL-6-mediated cancer metastasis. First, IL-6 promoted mRNA and protein expression of ICAM-1. Second, ICAM-1 siRNA significantly reduced IL-6-mediated cell motility. Third, SCC4/IL-6 shRNA cells showed a greater reduction in migration and ICAM-1 expression than did SCC4/control shRNA cells. Although ICAM-1 was reportedly associated with the cell motility of OSCC cells, we demonstrated that ICAM-1 is a downstream effector in IL-6-increased metastasis of OSCC. Adhesion molecules that are expressed in cancer cells can attract inflammatory cells such as macrophages or lymphocytes, which release trophic factors to enhance cancer cell survival and destabilize the tumor environment [33,34]. For example, tumor-associated macrophages, the major component of infiltrating inflammatory cells, release growth and angiogenic factors that stimulate tumor cell proliferation, promote angiogenesis, and even favor invasion and metastasis [34]. However, in our study, we were unable to examine whether human OSCC with high ICAM-1 expression aggregated more leukocytes and macrophages. Therefore, further studies are necessary to confirm this hypothesis regarding human OSCC.

Syk activation has been reported to be regulated by multiple mechanisms, including phosphorylation and interactions with various proteins that are involved in regulating gene expression [35]. We demonstrated that the Syk inhibitor antagonized the IL-6-mediated potentiation of ICAM-1 expression and cell migration. In addition, transfection of cells with Syk siRNA reduced IL-6-mediated potentiation of ICAM-1 expression and cell motility. These data suggest that the Syk pathway is required for IL-6-induced cell migration and ICAM-1 expression in OSCC cells. A Syk-dependent JNK pathway was shown to be involved in CCN4-induced gene expression [28]. In the current study, we found that IL-6 enhanced Syk and JNK phosphorylation. Pretreatment of cells with either IL-6R or the Syk inhibitor reduced IL-6-promoted JNK phosphorylation. Thus, our results indicate that IL-6 upregulates ICAM-1 expression and cell migration in human OSCC cells via the IL-6R, Syk, and JNK signaling pathways.

The Jun and Fos transcription factor families bind to the AP-1 sequence. These nuclear proteins interact with the AP-1 site as Jun homodimers or Jun-Fos heterodimers that are formed by protein dimerization through their leucine zipper motifs. In this study, we found that IL-6 promoted c-Jun phosphorylation and translocation into the nucleus. In addition, IL-6-mediated cell migration and ICAM-1 expression were abolished by c-Jun siRNA in human OSCC cells. Therefore, c-Jun activation is mediated by IL-6-increased cancer metastasis and ICAM-1 expression. Furthermore, IL-6 increased the binding of c-Jun to the AP-1 element within the ICAM-1 promoter, as shown by ChIP. Pretreatment of cells with IL-6R mAb, the Syk inhibitor, and SP600125 abolished the binding of c-Jun to the AP-1 element. These results indicate that IL-6 acts through the IL-6R, Syk, JNK, c-Jun, and AP-1 pathways to induce metastasis and ICAM-1 expression in human OSCC cells. In the current study, IL-6R, Syk, JNK, and c-Jun inhibitor all reduced IL-6-induced cell migration in three OSCC cell lines, indicating that IL-6R, Syk, JNK, and c-Jun pathways are involved in IL-6-mediated metastasis in these cells. However, to examine whether these pathways are activated after IL-6 treatment, we used the most widely used OSCC cells (SCC4) to represent the IL-6 effects.

## 4. Experimental Section

### 4.1. Materials

Protein A/G beads, anti-mouse and anti-rabbit horseradish peroxidase-conjugated IgG, rabbit polyclonal antibodies specific for ICAM-1, IL-6, Syk, p-Syk, p-JNK, JNK, p-c-Jun, c-Jun, and β-actin, and the IL-6 short hairpin RNA (shRNA), and control shRNA plasmids were purchased from Santa Cruz Biotechnology (Santa Cruz, CA, USA). Recombinant human IL-6 and IL-6R antibodies were purchased from R&D Systems (Minneapolis, MN, USA). The JNK dominant-negative mutant was provided by Dr. Michael Karin (University of California, San Diego, CA, USA). The luciferase assay kit was purchased from Promega (Madison, WI, USA). All other chemicals were purchased from Sigma-Aldrich (St. Louis, MO, USA).

### 4.2. Cell Culture

Human OSCC cell lines (SCC4, SAS, and CAL27 cells) were purchased from the American Type Culture Collection (Manassas, VA, USA). The cells were maintained in Dulbecco’s modified Eagle’s medium (DMEM; Gibco, Grand Island, NY, USA) supplemented with 20 mM HEPES [4-(2-hydroxyethyl)-1-piperazineethanesulfonic acid, Gibco, Grand Island, NY, USA] and 10% heat-inactivated fetal calf serum (FCS), 2 mM glutamine, penicillin (100 U/mL), and streptomycin (100 μg/mL) at 37 °C with 5% CO_2_.

IL-6 shRNA-expressing cells were selected with puromycin. Surviving cells were collected and expanded to prepare clonal cell populations. For monolayer growth curves, 10^4^ cells were plated in 6-well plates and grown for 1–6 days. Cells were trypsinized and cell numbers were counted [36]. Cells were cultured in DMEM supplemented with 20 mM HEPES and 10% heat-inactivated FCS, 2 mM glutamine, penicillin (100 U/mL), and streptomycin (100 μg/mL) at 37 °C with 5% CO_2_.

### 4.3. Migration Assay

The migration assay was performed using Transwell inserts (Costar, Corning, New York, NY, USA; pore size, 8 μm) in 24-well dishes. Before the migration assay was performed, cells were pretreated for 30 min with different concentrations of inhibitors, including the Syk inhibitor, SP600125, tanshinone, curcumin, and vehicle control [0.1% dimethyl sulfoxide (DMSO)]. Approximately 1 × 10^4^ cells in 200 μL of serum-free medium were placed in the upper chamber, while 300 μL of serum-free medium containing IL-6 was placed in the lower chamber. The plates were incubated for 24 h at 37 °C in 5% CO_2_. Next, the cells were fixed in 3.7% formaldehyde solution for 15 min and stained with 0.05% crystal violet in phosphate-buffered saline (PBS) for 15 min. Cells on the upper side of the filters were removed using cotton-tipped swabs and the filters were washed with PBS. Cells on the underside of the filters were examined and counted under a microscope. Each clone was plated in triplicate in each experiment; each experiment was repeated at least 3 times. The number of migrating cells in each experiment was adjusted by the cell viability assay to correct for proliferation effects of IL-6 treatment (corrected migrating cell number = counted migrating cell number/percentage of viable cells) [37].

### 4.4. Wound-Healing Migration Assay

For the wound-healing migration assay, cells were seeded into 12-well plates at a density of 1 × 10^5^ cells/well in culture medium. Twenty-four hours after seeding, the confluent monolayer of culture was scratched using a fine pipette tip, and migration was visualized using a microscope with magnification. The rate of wound closure was observed at the indicated time.

### 4.5. Quantitative Real-Time Polymerase Chain Reaction (PCR)

Total RNA was extracted from OSCC cells using a TRIzol kit (Invitrogen, Carlsbad, CA, USA). The reverse transcription reaction was performed using 2 μg of total RNA that was reverse-transcribed into cDNA using an oligo (dT) primer (Invitrogen, Carlsbad, CA, USA) [38]. Quantitative real-time PCR (qPCR) analysis was carried out using TaqMan^®^ one-step PCR Master Mix (Applied Biosystems, Foster City, CA, USA). Two microliters of total cDNA mixtures was added per 25-μL reaction with sequence-specific primers and Taqman^®^ probes. All target gene primers and probes were purchased commercially (β-actin was used as an internal control) (Applied Biosystems). qPCR assays were performed in triplicate (1 independent RNA sample per treatment) using a StepOnePlus sequence detection system (Applied Biosystems, Foster City, CA, USA). Cycling conditions were as follows: polymerase activation at 95 °C for 10 min followed by 40 cycles at 95 °C for 15 s and 60 °C for 60 s. The threshold was set above the no-template control background and within the linear phase of the target gene amplification to calculate the cycle number at which the transcript was detected (denoted C_T_).

### 4.6. Western Blot Analysis

Cellular lysates were prepared as described previously [39]. Proteins were resolved by sodium dodecyl sulfate polyacrylamide gel electrophoresis (SDS-PAGE) and transferred to Immobilon polyvinyldifluoride (PVDF) membranes (Millipore, Billerica, MA, USA). The blots were blocked with 4% bovine serum albumin (BSA) for 1 h at room temperature and then probed with rabbit anti-human antibodies against ICAM-1, p-Syk, Syk, p-JNK, JNK, p-c-Jun, or c-Jun (1:1000) for 1 h at room temperature. After 3 washes, the blots were incubated with a donkey anti-rabbit peroxidase-conjugated secondary antibody (1:1000) for 1 h at room temperature. Blots were visualized using enhanced chemiluminescence and Kodak X-OMAT LS film (Eastman Kodak, Rochester, NY, USA).

### 4.7. Transfection of Small Interfering RNAs (siRNAs) or Mutant

ON-TARGETplus siRNA targeting ICAM-1, Syk, c-Jun, and controls were purchased from Dharmacon Research (Lafayette, CO, USA). Transient transfection of siRNAs (100 nM) or dominant-negative mutants (0.5 μg) was carried out using DharmaFECT 1 transfection reagent or Lipofectamine 2000 (Invitrogen) according to the manufacturer’s instructions, respectively.

### 4.8. Flow Cytometric Analysis

Human OSCC cells were plated in 6-well dishes. The cells were then washed with PBS and detached with trypsin at 37 °C. Cells were fixed for 10 min in PBS containing 1% paraformaldehyde. After rinsing in PBS, the cells were incubated with mouse anti-human antibody against ICAM-1 (1:100) for 1 h at 4 °C. Cells were then washed again and incubated with fluorescein isothiocyanate (FITC)-conjugated goat anti-rabbit secondary IgG (1:100; Leinco Tec. Inc.; St. Louis, MO, USA) for 45 min and analyzed by flow cytometry using FACS Calibur and CellQuest software (BD Biosciences, Franklin Lakes, NJ, USA).

### 4.9. Immunofluorocytochemistry

SCC4 cells were cultured on 12-mm coverslips. After treatment with IL-6, cells were fixed with 4% paraformaldehyde at room temperature. Thirty minutes later, 4% nonfat milk in PBS containing 0.5% Triton X-100 was added to the cells. The cells were then incubated with rabbit anti-c-Jun (1:100) and FITC-conjugated goat anti-rabbit secondary antibody (1:500; Leinco Technology Inc., St. Louis, MO, USA) for 1 h, successively. FITC was detected using a Zeiss fluorescence microscope.

### 4.10. Chromatin Immunoprecipitation (ChIP)

ChIP was performed as described previously [13]. DNA was immunoprecipitated using an anti-c-Jun antibody, extracted, purified, and resuspended in H_2_O. Immunoprecipitated DNA was amplified by PCR using the following primers: 5′-AGACCTTAGCGCGGTGTAGA-3′ and 5′-GCGACTCGAGGAGACGATGA-3′. PCR products were resolved by 1.5% agarose gel electrophoresis and visualized using ultraviolet light.

### 4.11. Statistical Analysis

Data are presented as the mean ± standard error of the mean (SEM). Statistical analysis between 2 samples was performed using the Student’s *t*-test. Statistical comparisons of more than 2 groups were performed using one-way analysis of variance with Bonferroni’s post-hoc test. In all cases, *p* < 0.05 was considered significant.

## 5. Conclusions

We have presented a molecular mechanism of IL-6-induced migration of human OSCC cells that occurs through the upregulation of ICAM-1. IL-6 increases ICAM-1 expression through the IL-6R, Syk, JNK, and AP-1 signaling pathways and induces tumor migration.
